# Application of an Electronic Nose Instrument to Fast Classification of Polish Honey Types

**DOI:** 10.3390/s140610709

**Published:** 2014-06-18

**Authors:** Tomasz Dymerski, Jacek Gębicki, Waldemar Wardencki, Jacek Namieśnik

**Affiliations:** 1 Department of Analytical Chemistry, Chemical Faculty, Gdansk University of Technology, 11/12 G. Narutowicza Str., 80-233 Gdańsk, Poland; E-Mails: waldemar.wardencki@pg.gda.pl (W.W.); jacek.namiesnik@pg.gda.pl (J.N.); 2 Department of Chemical and Process Engineering, Chemical Faculty, Gdansk University of Technology, 11/12 G. Narutowicza Str., 80-233 Gdańsk, Poland; E-Mail: jacek.gebicki@pg.gda.pl

**Keywords:** electronic nose, honey, PCA, LDA, cluster analysis

## Abstract

The paper presents practical utilization of an electronic nose prototype, based on the FIGARO semiconductor sensors, in fast classification of Polish honey types—acacia flower, linden flower, rape, buckwheat and honeydew ones. A set of thermostating modules of the prototype provided gradient temperature characteristics of barbotage-prepared gas mixtures and stable measurement conditions. Three chemometric data analysis methods were employed for the honey samples classification: principal component analysis (PCA), linear discriminant analysis (LDA) and cluster analysis (CA) with the furthest neighbour method. The investigation confirmed usefulness of this type of instrument in correct classification of all aforementioned honey types. In order to provide optimum measurement conditions during honey samples classification the following parameters were selected: volumetric flow rate of carrier gas—15 L/h, barbotage temperature—35 °C, time of sensor signal acquisition since barbotage process onset—60 s. Chemometric analysis allowed discrimination of three honey types using PCA and CA and all five honey types with LDA. The reproducibility of 96% of the results was within the range 4.9%–8.6% CV.

## 1. Introduction

Food safety and suitable quality of food products are fundamental issues that must be addressed at each stage of its production and distribution in order to avoid health and life hazards of potential consumers [1–3]. As a result a number of food monitoring and control methods have been developed [4–12]. Headspace analysis can be a source of valuable information on the properties of particular food products [13–18]. Such an approach is frequently utilized in many food producing facilities. Typically it takes advantage of sensory analysis, which exhibits numerous shortcomings due to the limitations of human senses [19–22]. That is why the authors have made an attempt to evaluate particular food product, honey, with a selected instrumental technique.

Current changes of the European Union regulations enforce the requirement to label food products with the facts concerning their quality, properties, origin and ingredients. Detailed descriptions on food product labels will increase consumer safety in a number of ways, mainly by being able to warn the consumer of the presence of any product s/he is allergic to. At present the legal issues concerning bee honey intended for the Polish market and for export are gathered in the Polish standard PN-88/ A-77626 [23]. It contains organoleptic requirements, including the methods of honey odour investigation, and points out the content of dominant pollen in honey deposits as the method of classification of honey type and origin. Both methods mentioned in the standard make it possible to classify honey, however, they do not provide unequivocal information about honey origin [23]. Moreover, they are time-consuming, especially the pollen analysis method, which consists in determination of the type of pollen grains present in a sample of honey and counting of dominant pollen observed under an optical microscope. A solution of this problem can be implementation of an instrumental technique. There are two main approaches to analysis of volatile fractions using instrumental techniques: the first one consists in separation of the sample components and their identification with gas chromatography technique [17,24–30], the second one is based on comprehensive analysis of volatile fraction without its separation into individual components. The second method requires application of electronic nose instruments, which unlike chromatographic techniques allow a significant shortening of the time needed for a single analysis, non-destructive interaction with the sample and skipping or substantial shortening of the sample preparation stage [2,31–40]. Examples of electronic nose application to investigation of honey matrices are provided in [33,41]. Another approach enabling discrimination of different honey types is the utilization of electronic tongues [42–45].

The intention of the authors was verification of the possibility of application of an electronic nose prototype to fast determination of the origin of Polish honey types. The device was equipped with a module of six semiconductor sensors by FIGARO Co. and a pneumatic system enabling the barbotage process of dissolved honey samples. The cost of the prototype was several times lower than that of commercially available electronic nose instruments. Currently and for different reasons the Polish food industry does not take full advantage of modern instrumental techniques. The problem of quality and botanic (raw material) origin control of honey available on the Polish market is still unsolved, although it is of significant importance from the economical and consumer safety standpoint. As mentioned before, the current honey investigation methods are time-consuming, which is reflected in the high cost of a single analysis. Fast and correct identification of honey type is going to determine the price of this product, which is higher in case of monofloral honey. An important issue is also not to mislead potential customers. The information provided on the label must be in accordance with the facts. Hence, elaboration of a relatively cheap device dedicated to the problem described in this paper may result in its practical application in the Polish honey industry as a tool for routine tests in Polish national laboratories.

## 2. Materials and Methods

### 2.1. Instrumentation

The main element of the measurement set-up was the electronic nose prototype. It was equipped with a module consisting of six semiconductor sensors (TGS 880, TGS 825, TGS 826, TGS 822, TGS 2610, TGS 2602; Table 1) manufactured by FIGARO Co. (Osaka, Japan) and with thermostating modules for the gas mixture obtained via the barbotage process to prevent condensation of gas sample components inside the measurement set-up as well as providing stable and reproducible sensors response signals [46].

**Figure 1.**
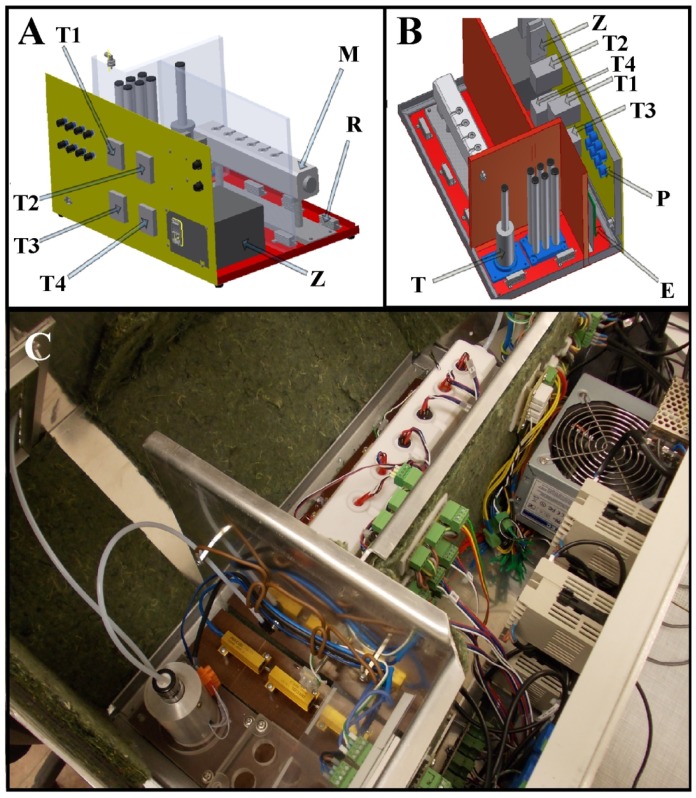
Design of the electronic nose prototype; **A** and **B**—screen-shots from Autodesk^®^ Inventor^®^ 3D software: T1, T2, T3, T4—modules controlling temperature of particular elements of electronic nose prototype, Z—power supply 230 VAC, R—heating element, M—sensors module, P—potentiometer, E—integrated circuit responsible for preliminary processing of TGS sensors response signals, T—heating jacket controlling temperature of sample during barbotage; **C**—photo—top view.

Precise maintenance of the sample's temperature makes it possible to determine the concentration of an analyte in gas phase precisely. Relative humidity of the prepared gas mixtures was adjusted with the sample's temperature as well as with the inert gas flow rate and was from 86% to 91%. Higher sample relative humidity results in an increase in sensor sensitivity and in enhancement of metrological parameters of the electronic nose. In order to provide constant (adjustable) gas sample temperatures and relatively high humidity four thermostating modules were applied. They utilized AT-503 temperature controllers by ANLY Co. (New Taipei City, Taiwan) and Pt100 temperature sensors: T1—module controlling the temperature of the heating jacket for the samples, T2—module controlling the temperature of the sample chamber, T3—module controlling the temperature in the sensor chamber, T4—module controlling the temperature of the module with the sensors. Application of four thermostating modules allows one to obtain a desired and constant temperature gradient (T1 < T2 < T3 < T4), which prevents condensation of water from gas samples on their way from the sample chamber to the output of the electronic nose system (Figure 1). In the discussed siituation the temperature gradient is characterised by the following dependences: T2 = T1 + 2 °C, T3 = T2 + 5 °C, T4 = T3 + 5 °C, where T1 ε <10 °C; 50 °C>. In order to ensure correct operation of the modules the electronic nose instrument was equipped with an appropriate casing made of heat-insulating materials. The casing was divided into sample chamber, sensor chamber and electronic systems chamber. Each wall separating the chambers was heat-insulated. The structure of the casing is presented in Figure 1.

**Figure 2.**
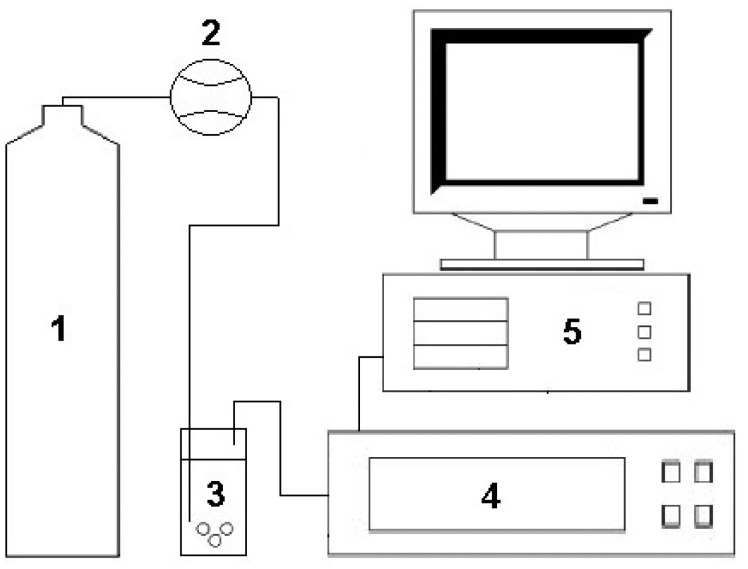
Experimental set-up for analysis of volatile fraction of Polish honey consisting of: 1—bottle with carrier gas; 2—flow meter; 3—scrubber; 4—electronic nose prototype; 5—PC.

A miniaturized electronic circuit conditioned the output signals from the semiconductor sensors and converted them into digital form. Application of this circuit enabled lowering the limit of detection of the sensors by one order of magnitude [47]. Compressed air of N5.0 purity (Linde Gaz Polska Sp. z o.o., Kraków, Poland) was the carrier gas. The main elements of the measurement set-up are a carrier gas bottle with a bottle reducer with metal membrane dedicated for non-corrosive gases made by IHW Group (Berlin, Germany), a 2150 series flow meter (Tecfluid, Barcelona, Spain). The electronic nose prototype is connected using Teflon tubes of 4 mm diameter. Data acquisition was performed with a PC-class computer. The abovementioned measurement set-up has already been described in [47,48] and is presented in Figure 2.

**Figure 3.**
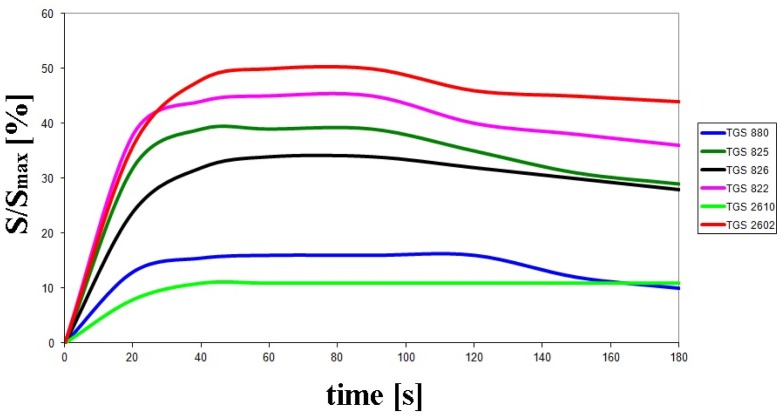
Exemplary course of sensors response signals *versus* time obtained during analysis of rape honey at optimum operation parameters of electronic nose prototype: barbotage temperature 35 °C, volumetric flow rate of carrier gas—15 L/h, time of sensor signal acquisition since barbotage process onset—60 s.

Figure 3 illustrates exemplary sensors response signals *versus* time recorded for the optimum operation conditions of the electronic nose prototype. The values presented on the vertical axis are expressed as a ratio of sensor signal to the full measurement range—S/S_max_ (the output signal was a digital one within the range from 0 to 14 bits).

### 2.2. Software for Chemometric Data Analysis

Interpretation of obtained data was performed with commercially available software SAS Enterprise 4.3 (SAS Co., Cary, NC, USA) with implemented PRINCOMP algorithm (for principal component analysis; PCA) and free software R being a part of Free Software Foundation (Free Software Foundation, Boston, MA, USA) utilized for linear discriminant analysis (LDA) and cluster analysis with the furthest neighbour method.

### 2.3. Object of Investigation

The investigations were conducted for five different types of Polish honey: acacia flower, linden flower, rape, buckwheat and honeydew ones. A total of 15 honey samples were analysed (three samples of each type of honey). The honey originated from various regions of Poland belonging to the following voivodeships: Pomorskie, Warmińsko-Mazurskie, Kujawsko-Pomorskie, Śląskie, Małopolskie. The honey was obtained from the Polish Beekeeping Society, which ensured its authenticity. The samples had been stored in dark room, at constant temperature of 20 °C for six months before they were investigated.

### 2.4. Preparation of Samples for Analysis

Preparation of the Polish honey samples consisted in weighing honey (1 g), placing it in a 15 mL vial and dilution with deionized water taken from a Milli-Q A10 device (Millipore Co., Billerica, MA, USA). The final volume of the sample was 5 mL. A total number of prepared samples was 825. The investigations were performed over 3 months.

## 3. Results and Discussion

Correct classification of Polish honey types required optimization of the electronic nose operation conditions. The parameters subjected to optimization included: temperature of barbotage process, volumetric flow rate of carrier gas, time of sensor signal acquisition since barbotage process onset. Section 3.1 presents the results of investigation on the barbotage temperature influence on honey discrimination abilities. The temperature of the barbotage process was the most important parameter in the entire optimization cycle. That is why the authors present step by step how the optimum barbotage temperature allowing the best discrimination of honey types was determined. Analysing Equation (1) describing dependence between analyte concentration in the gas phase and the barbotage process conditions one can notice that the concentration of a given component in gas phase is determined by a partition coefficient. The lower its value is, the higher the concentration of the particular component in the gas phase is. The value of the partition coefficient depends on the thermodynamic equilibrium conditions between the liquid and gas phase. The main parameters influencing on the value of partition coefficient are type of analyte and temperature:

(1) $$c_{G}=\frac{c_{L0}}{K}\exp\left(-\frac{Qt}{KV_{L}}\right)$$

where: *c_L_*_0_—initial analyte concentration in liquid phase, *c_G_*—instantaneous analyte concentration in gas phase, *Q*—volumetric flow rate of inert gas, *t*—time of barbotage, *V_L_*—volume of liquid phase, *K*—partition coefficient described by the Equation (2):

(2) $$K=\frac{c_{Lr}}{c_{GR}}$$

where: *c_LR_*—analyte concentration in liquid phase being in thermodynamic equilibrium with gas phase, *c_GR_*—analyte concentration in gas phase being in thermodynamic equilibrium with liquid phase.

Section 3.1 contains an analysis of the influence of barbotage temperature within the range from 15 °C to 35 °C based on the chemometric analysis results using PCA, LDA and CA. Section 3.2 presents summary information about the remaining parameters subjected to optimization. The best conditions of the electronic nose prototype operation enabling correct discrimination of Polish honey types were determined.

Calculation of the *Coefficient of Variation* (CV) coefficients made it possible to check the reproducibility of the obtained results. The reproducibility of 96% of the results was within the range 4.9%–8.6% CV and was calculated based on five analyses (the first one and four repetitions) for every sample of honey. Moreover, these five analyses for each sample allowed chemometric analysis using averaged measurement data (each point in multidimensional data space was an average of five measurements). This provided clear graphical presentation of the analyses results, especially dendrograms for cluster analysis.

### 3.1. Investigation of the Barbotage Temperature Influence

**Figure 4.**
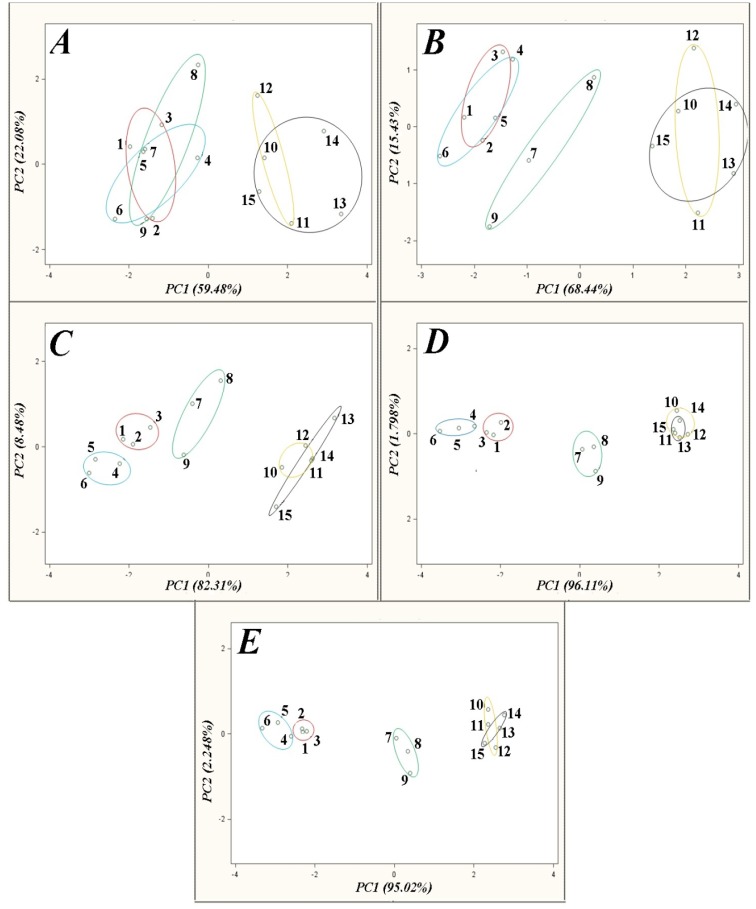
PCA results for honey samples obtained for barbotage temperature: **A**—15 °C, **B**—20 °C, **C**—25 °C, **D**—30 °C, **E**—35 °C; points: 1–3—acacia flower honey (marked in red), 4–6—linden flower honey (marked in blue), 7–9—rape honey (marked in green), 10–12—buckwheat honey (marked in yellow), 13–15—honeydew (marked in black).

**Figure 5.**
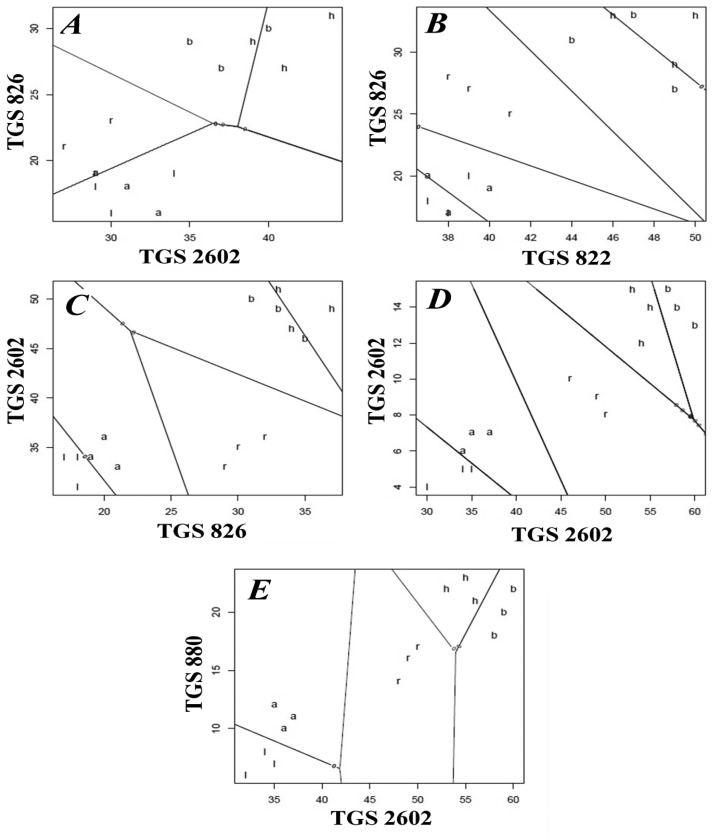
Classification of honey samples using LDA for the following barbotage temperatures: **A**—15 °C, **B**—20 °C, **C**—25 °C, **D**—30 °C, **E**—35 °C; a—acacia flower honey, l—linden flower honey, r—rape honey, b—buckwheat honey, h—honeydew.

**Figure 6.**
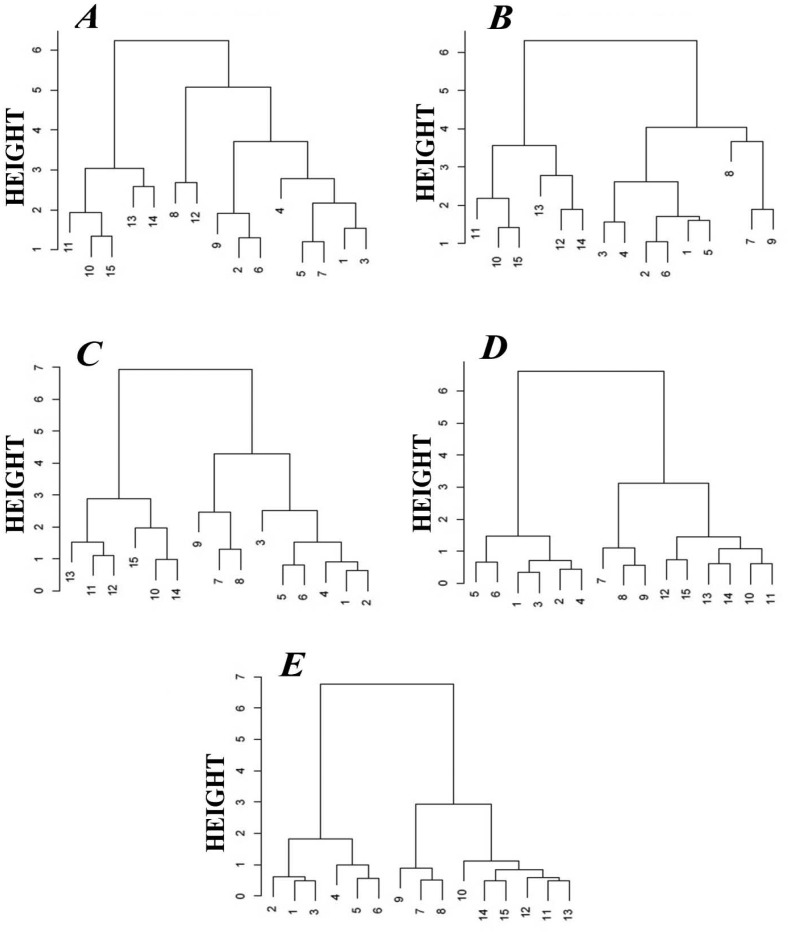
Classification of honey samples using CA for the following barbotage temperatures: **A**—15 °C, **B**—20 °C, **C**—25 °C, **D**—30 °C, **E**—35 °C; 1–3—acacia flower honey, 4–6—linden flower honey, 7–9—rape honey, 10–12—buckwheat honey, 13–15—honeydew.

Figures 4, 5 and 6 present the influence of barbotage temperature changes on the results of the following analyses of honey samples: principal component analysis (PCA), linear discriminant analysis (LDA) and cluster analysis (CA) with the farthest neighbour method. The remaining parameters of the barbotage process and of the electronic nose prototype operation were held constant during each analysis and were as follows: sample volume—5 mL, volumetric flow rate of carrier gas—15 L/h, time of sensor signal acquisition since barbotage process onset—60 s. Change of temperature of barbotage process was accomplished via regulation of the temperature of a heating jacket with the samples within the range from 15 °C to 35 °C.

The PCA results presented in Figure 4 reveal a significant improvement of the points groups separation on the PC1PC2 plane with an increase in barbotage temperature. This dependence can result from the fact that a richer profile of volatile components in the gas phase is obtained at higher barbotage temperatures. Comparison of the sensors' response time corresponding to analysis of the gas mixtures richer in components shows bigger differences in volatile fraction composition, which consequently results in a bigger differentiation in localization of the points groups on the PCA plot. Moreover, an increase in barbotage temperature is associated with an increase in the result precision (dispersion of the points within particular group is smaller). In Figure 4A the highest dispersion of points corresponds to particular groups. The points corresponding to the group of acacia flower, linden flower, rape honey and separately buckwheat honey, honeydew are located in overlapping regions of the PCA plot. Both regions are relatively close to each other on the PCA plot. An increase in barbotage temperature by 5 °C (Figure 4B) results in separation of the points for rape honey from the remaining points groups on the plot. However, the dispersion of points, for this way of analysis, is similar to the previous case. Another increase in barbotage temperature by 5 °C (Figure 4C) causes a decrease in the points dispersion and results in separation of the points groups associated with acacia flower and linden flower honey on the PCA plot.

An increase in barbotage temperature to 30 °C (Figure 4D) yields even smaller points dispersion within particular groups. A distance of the points group of rape honey significantly increased with respect to the remaining points groups. Rising barbotage temperature from 30 °C to 35 °C results in small improvement of points separation on the PCA plot as compared to the previous plot. Points distribution within particular groups and the distances between the points groups are similar to the PCA result obtained for the barbotage temperature of 30 °C. However, the points group for acacia flower honey (the points 1–3) is characterized by the smallest points dispersion. Accordingly, the PCA result presented in Figure 4E was considered the best with respect to separation of the points groups representing different types of honey.

Considering the problem of classification (discrimination) between the samples of different types of honey linear discriminant analysis (LDA) was employed, too. Classifier's evaluation was the averaged evaluation of ten cross-validations by variable addition method. As a result of this evaluation the best pairs of variables (sensor selection) were chosen and taken for LDA. Figure 5A shows that application of the classifier did not allow discrimination of any of the five honey types. An increase in barbotage temperature to 20 °C (Figure 5B) resulted in the fact that the LDA method allowed correct classification of rape honey, similarly as in the case of PCA presented in Figure 5B. Further increase in barbotage temperature by 5 °C enabled classification of acacia flower and linden flower honey into two separate groups. At this stage the LDA method as well as the PCA one made it possible to discriminate three types of honey. In the case of the LDA results presented in Figures 5D,E there is classification of all five honey types, which had not been possible with the PCA method. Comparing the classification based on both chemometric methods it can be stated that LDA is a better tool for evaluation of the botanical origin of honey while using the presented electronic nose prototype.

Another method of graphical presentation of the information contained in a matrix of distance between the objects is cluster analysis. This method utilizes a dendrogram to present hierarchical grouping of objects set. There are several ways to construct the dendrogram based on the nearest neighbour method, centroid method, however, the widest application in the field of cluster analysis is found by the furthest neighbour method, which emphasizes difference between the elements of analyzed set. In this paper we also employed the classifier of unsupervised cluster analysis (“without teacher”) in order to compare its effectiveness with the LDA supervised classifier (“with teacher”). Figure 6 presents the dendrograms illustrating a degree of similarity between the honey samples. The dendrograms presented in Figures 6A,b do not allow correct classification of the honey samples with respect to their origin. In Figure 6C one can clearly notice separated branches for rape honey (the points 7–9). The best classification properties are revealed by the dendrogram presented in Figure 4E because it allows discrimination of three honey types, namely acacia flower (1–3), linden flower (4–6) and rape (7–9) ones. The CA result shown in Figure 6D enabled discrimination of rape honey (7–9) only and it did not correlate with the LDA result obtained for barbotage temperature of 30 °C, which made it possible to discriminate all types of honey. Comparing PCA, LDA and CA it is evident that the barbotage temperature of 35 °C provided the best results as far as classification of honey with respect to its botanical origin is concerned.

### 3.2. Summary of the Optimized Operation Parameters of the Electronic Nose Prototype Influencing on the Results of Chemometric Analysis

Table 2 presents the results of correctly discriminated honey types via the three methods of chemometric analysis used depending on the parameters subjected to an optimization process. A total of 825 analyses were performed (15 honey samples × 5 repetitions × 11 conditions of prototype electronic nose operation).

It can be noticed that the highest number of correctly discriminated honey types is associated with the chemometric methods, for which the operation parameters of the electronic nose prototype were as follows: volumetric flow rate of carrier gas 15 L/h, barbotage temperature 35 °C, time of sensor signal acquisition since barbotage process onset 60 s. Figures 4E, 5E and 6E in Section 3.1 describe this situation. Application of PCA and CA chemometric analysis for the optimum operation conditions of the electronic nose prototype allowed discrimination of three honey types, namely acacia flower, linden flower and rape ones. In these conditions LDA method made it possible to discriminate all types of honey.

## 4. Summary

The aim of the investigation was to verify if an electronic nose device prototype can be successfully employed for fast classification of different types of Polish honey. The prototype was equipped with a module consisting of six semiconductor sensors by FIGARO Co. and a pneumatic system enabling the barbotage of dissolved honey samples. A miniaturized electronic circuit constituted one of the main elements of the electronic nose prototype and allowed obtaining sensors output signals in digital form. Implementation of this circuit made it possible to decrease the limit of detection of the semiconductor sensors by one order of magnitude. Application of a set of thermostating modules [46] providing gradient temperature characteristics of gas mixtures prepared via the barbotage process and stable conditions of measurement contributed to reproducibility of 96% of results, in the range 4.9%–8.6% CV. Time necessary for a single analysis of honey sample was 60 s. The cost of design and assembly of this dedicated device was several times lower than that of commercial electronic nose instruments.

## 5. Conclusions

Depending on the chemometric method applied the prototype of electronic nose provided different classification capability with respect to honey of various botanical origins. The electronic nose instrument allowed complete discrimination between the types of Polish honey samples using the LDA classifier. In the case of the PCA and CA analysis it was possible to discriminate between a maximum of three types of honey, namely acacia flower, linden flower and rape ones. The following measurement parameters were the optimum ones for the barbotage process, the electronic nose prototype used and for all three chemometric methods applied: sample volume—5 mL, volumetric flow rate of carrier gas—15 L/h, barbotage temperature—35 °C, time of sensor signal acquisition since barbotage process onset 60 s. This could have been caused by the fact that the main influence on discrimination resulted from the total concentration of the components of the volatile fraction of honey. Based on a literature report [23] and the presented results obtained via the GC × GC-TOFMS technique it is known that the volatile component profiles of acacia flower and linden flower honey are poorer than rape honey one. Buckwheat honey and honeydew are characterized by the richest profile of volatile components. The lowered limit of detection of the semiconductor sensors applied in the electronic nose prototype was still relatively high (for some sensors it was *ca.* 0.1 ppm v/v). In spite of this fact, the LDA classifier enabled discrimination between all the types of Polish honey investigated.

Summarizing, the authors of the paper believe that in future the electronic nose prototype can be a useful tool for fast and objective classification of Polish honey types with respect to their origin. The results presented may be complementary to the Polish standard PN-88/A-77626 and may find application in industrial practice.

## Figures and Tables

**Table 1.** Characteristics of TGS sensors utilized in the prototype of electronic nose.

**Sensor**	**Targeted Gases**	**Smell Description**
TGS 880	volatile vapors from food	food (while cooking)
TGS 825	hydrogen sulfide	rotten egg, sulfurous smells
TGS 826	ammonia	old rotten urine and fish (sharp, penetrating, and irritating smell)
TGS 822	alcohol, xylene, toluene, other VOC	aromatic solvents, wood fermentation, alcoholic beverages
TGS 2610	general hydrocarbons	smell of ripening fruits
TGS 2602	ammonia, hydrogen sulfide,ethanol, toluene	a wide range of smell: from rotten fish to wood fermentattion

**Table 2.** Influence of operation parameters of electronic nose prototype on results of chemometric analysis aimed at discrimination of Polish honey types.

**Parameters Subjected to Optimization**			**Number of Honey Types Correctly Discriminated via Chemometric Analysis**		

**Volumetric flow rate of carrier gas [L/h]**	**Barbotage temperature [°C]**	**Time of sensor signal acquisition since barbotage process onset [s]**	**PCA**	**LDA**	**CA**
5	35	60	1	0	1
10	35	60	1	3	1
**15**	**35**	**60**	**3**	**5**	**3**
15	30	60	3	5	1
15	25	60	3	3	1
15	20	60	1	1	0
15	15	60	0	0	0
15	35	20	1	1	0
15	35	90	3	5	1
15	35	120	1	1	1
15	35	180	1	1	0

## References

[1] Panigrahi S., Balasubramanian S., Gu H., Logue C., Marchello M. (2006). Neural-network-integrated electronic nose system for identification of spoiled beef. LWT Food Sci. Technol..

[2] Capone S., Epifani M., Quaranta F., Siciliano P., Taurino A., Vasanelli L. (2001). Monitoring of rancidity of milk by means of an electronic nose and a dynamic PCA analysis. Sens. Actuators B.

[3] Blixt Y., Borch E. (1999). Using an electronic nose for determining the spoilage of vacuum-packaged beef. Int. J. Food Microbiol..

[4] Berna A. (2010). Metal oxide sensors for electronic noses and their application to food analysis. Sensors.

[5] Peris M., Escuder-Gilabert L. (2009). A 21st century technique for food control: Electronic noses. Anal. Chim. Acta.

[6] Lachenmeier D.W., Richling E., López M.G., Frank W., Schreier P. (2005). Multivariate analysis of FTIR and ion chromatographic data for the quality control of tequila. J. Agric. Food Chem..

[7] Steine C., Beaucousin F., Siv C., Peiffer G. (2001). Potential of semiconductor sensor arrays for the origin authentication of pure Valencia orange juices. J. Agric. Food Chem..

[8] Berna A.Z., Trowell S., Cynkar W., Cozzolino D. (2008). Comparison of metal oxide-based electronic nose and mass spectrometry-based electronic nose for the prediction of red wine spoilage. J. Agric. Food Chem..

[9] Moio L., Ugliano M., Genovese A., Gambuti A., Pessina R., Piombino P. (2004). Effect of antioxidant protection of must on volatile compounds and aroma shelf life of Falanghina (*Vitis vinifera* L.) wine. J. Agric. Food Chem..

[10] Cortes M.B., Moreno J.J., Zea L., Moyano L., Medina M. (1999). Response of the aroma fraction in sherry wines subjected to accelerated biological aging. J. Agric. Food Chem..

[11] Marsili R.T. (2000). Shelf-life prediction of processed milk by solid-phase microextraction, mass spectrometry, and multivariate analysis. J. Agric. Food Chem..

[12] Benedetti S., Sinelli N., Buratti S., Riva M. (2005). Shelf life of Crescenza cheese as measured by electronic nose. J. Dairy Sci..

[13] Reinhard H., Sager F., Zoller O. (2008). Citrus juice classification by SPME-GC-MS and electronic nose measurements. LWT Food Sci. Technol..

[14] Barié N., Bücking M., Rapp M. (2006). A novel electronic nose based on miniaturized SAW sensor arrays coupled with SPME enhanced headspace-analysis and its use for rapid determination of volatile organic compounds in food quality monitoring. Sens. Actuators B.

[15] Conde J.E., Garcia-Montelongo F., Rodriguez-Bencomo J.J., Pérez-Trujillo J.P. (2003). Determination of major compounds in sweet wines by headspace solid-phase microextraction and gas chromatography. J. Chromatogr. A.

[16] Rodriguez-Bencomo J.J., Conde J.E., Rodriguez-Delgado M.A., Garcia-Montelongo F., Pérez-Trujillo J.P. (2002). Determination of esters in dry and sweet white wines by headspace solid-phase microextraction and gas chromatography. J. Chromatogr. A.

[17] Monje M.C., Privat C., Gastine V., Nepveu F. (2002). Determination of ethylphenol compounds in wine by headspace solid-phase microextraction in conjunction with gas chromatography and flame ionization detection. Anal. Chim. Acta.

[18] Dymerski T.M., Chmiel T.M., Wardencki W. (2011). Invited review article: An odor-sensing system-powerful technique for foodstuff studies. Rev. Sci. Instrum..

[19] Siverts H.K., Holen B., Nicolays F., Ris E. (1999). Classification of French red wines according to their geographical origin by the use of multivariate analyses. J. Sci. Food Agric..

[20] Lobo A.P., Tascon N.F., Madera R.R., Valles B.S. (2005). Sensory and foaming properties of sparkling cider. J. Agric. Food Chem..

[21] Hernandez-Gomez L., Ubeda-Iranzo J., Gracia-Romero E., Briones-Perez A. (2005). Comparative production of different melon distillates: Chemical and sensory analyses. Food Chem..

[22] Mildner-Szkudlarz S., Jeleń H.H., Zawirska-Wojtasiak R. (2008). The use of electronic and human nose for monitoring rapeseed oil autoxidation. Eur. J. Lipid Sci. Technol..

[23] Dymerski T., Chmiel T., Mostafa A., Śliwińska M., Wiśniewska P., Wardencki W., Namieśnik J., Górecki T. (2013). Botanical and geographical origin characterization of polish honeys by headspace SPME-GC×GC-TOFMS. Curr. Org. Chem..

[24] Diéguez S.C., Díaz L.D., Luisa M., de la Peña G., Gómez E.F. (2002). Variation of volatile organic acids in spirits during storage at low and room temperatures. LWT—Food Sci. Technol.

[25] Gewu W. (1997). Identification of character impact odorants of different white wine varieties. J. Agric. Food Chem..

[26] Cortés S., Gil L.M., Fernández E. (2005). Volatile composition of traditional and industrial Orujo spirits. Food Control.

[27] Plutowska B., Wardencki W. (2008). Application of gas chromatography–olfactometry (GC–O) in analysis and quality assessment of alcoholic beverages—A review. Food Chem..

[28] Wardencki W., Chmiel T., Dymerski T., Biernacka P., Plutowska B. (2009). Application Of gas chromatography, mass spectrometry and olfactometry for quality assessment of selected food products. Ecol. Chem. Eng. S.

[29] Plutowska B., Chmiel T., Dymerski T., Wardencki W. (2011). A headspace solid-phase microextraction method development and its application in the determination of volatiles in honeys by gas chromatography. Food Chem..

[30] Wardencki W., Chmiel T., Dymerski T., Biernacka P. (2009). Instrumental techniques used for assessment of food quality. Proc. ECOpole.

[31] Bhattacharyya N., Seth S., Tudu B., Tamuly P., Jana A., Ghosh D., Bandyopadhyay R., Bhuyan M. (2007). Monitoring of black tea fermentation process using electronic nose. J. Food Eng..

[32] Ampuero S., Bosset J.O. (2003). The electronic nose applied to dairy products: A review. Sens. Actuators B.

[33] Ampuero S., Bogdanov S., Bosset J.O. (2004). Classification of unifloral honeys with an MS-based electronic nose using different sampling modes: SHS, SPME and INDEX. Eur. Food Res. Technol..

[34] Aleixandre M., Lozano J., Gutiérrez J., Sayago I., Fernández M.J., Horrillo M.C. (2008). Portable e-nose to classify different kinds of wine. Sens. Actuators B.

[35] García M., Aleixandre M., Gutiérrez J., Horrillo M. (2006). Electronic nose for wine discrimination. Sens. Actuators B.

[36] García M., Aleixandre M., Gutiérrez J., Horrillo M.C. (2006). Electronic nose for ham discrimination. Sens. Actuators B.

[37] Guadarrama A., Ferna J., Souto J., de Saja J. (2001). Discrimination of wine aroma using an array of conducting polymer sensors in conjunction with solid-phase micro-extraction (SPME) technique. Sens. Actuators B.

[38] Guadarrama A., Fernández J., Íñiguez M., Souto J., de Saja J. (2000). Array of conducting polymer sensors for the characterisation of wines. Anal. Chim. Acta.

[39] Martí M.P., Busto O., Guasch J. (2004). Application of a headspace mass spectrometry system to the differentiation and classification of wines according to their origin, variety and ageing. J. Chromatogr. A.

[40] Schaller E., Bosset J., Escher F. (1998). “Electronic Noses” and Their Application to Food. LWT Food Sci. Technol..

[41] Benedetti S., Mannino S., Sabatini A.G., Marcazzan G.L. (2004). Original article electronic nose and neural network use for the classification of honey. Apidologie.

[42] Ulloa P.A., Guerra R., Cavaco A.M., Rosa da Costa A.M., Figueira A.C., Brigas A.F. (2013). Determination of the botanical origin of honey by sensor fusion of impedance e-tongue and optical spectroscopy. Comput. Electron. Agric..

[43] Masnan M.J., Mahat N.I., Zakaria A., Shakaff A.Y.M., Adom A.H., Sa'ad F.S.A. (2012). Enhancing classification performance of multisensory data through extraction and selection of features. Procedia Chem..

[44] Gutiérrez J.M., Haddi Z., Amari A., Bouchikhi B., Mimendia A., Cetó X., del Valle M. (2013). Hybrid electronic tongue based on multisensor data fusion for discrimination of beers. Sens. Actuators B Chem..

[45] Dias L.A., Peres A.M., Vilas-Boas M., Rocha M.A., Estevinho L., Machado A.A.S.C. (2008). An electronic tongue for honey classification. Microchim. Acta.

[46] Dymerski T., Wardencki W., Gębicki J., Fijało C. (2012). Świątoniowski, B. Sposób Oceny Jakości Destylatu Rolniczego i Urządzenie do Oceny Jakości Destylatów Rolniczych. Polish Patent No. P..

[47] Dymerski T., Gębicki J., Wiśniewska P., Sliwińska M., Wardencki W., Namieśnik J. (2013). Application of the electronic nose technique to differentiation between model mixtures with COPD markers. Sensors.

[48] Dymerski T., Gębicki J., Wardencki W., Namieśnik J. (2013). Quality evaluation of agricultural distillates using an electronic nose. Sensors.

